# Lhermitte-Duclos disease presenting with positron emission tomography-magnetic resonance fusion imaging: a case report

**DOI:** 10.1186/1752-1947-6-76

**Published:** 2012-03-06

**Authors:** Ferdinando Calabria, Giovanni Grillea, Maddalena Zinzi, Manlio Barbarisi, Emanuele Siravo, Marcello Bartolo, Giampaolo Cantore, Claudio Colonnese, Cristina Grasso, Orazio Schillaci

**Affiliations:** 1 Department of Nuclear Medicine, IRCCS Neuromed, Pozzilli (IS), Italy; 2 Department of Neuroradiology, IRCCS Neuromed, Pozzilli (IS), Italy; 3 Department of Neurosurgery, II University of Naples, Italy; 4 Department of Neurosurgery, IRCCS Neuromed, Pozzilli (IS), Italy; 5 Department of Biopathology and Diagnostic Imaging, University 'Tor Vergata', Rome, Italy

## Abstract

**Introduction:**

Lhermitte-Duclos disease or dysplastic gangliocytoma of the cerebellum is an extremely rare tumor. It is a slowly enlarging mass within the cerebellar cortex. The majority of cases are diagnosed in the third or fourth decade of life.

**Case presentation:**

We report the case of a 37-year-old Caucasian woman who underwent positron emission tomography-computed tomography with fluorine-18-fluorodeoxyglucose for evaluation of a solitary lung node. No pathological uptake was detected in the solitary lung node but the positron emission tomography-computed tomography of her brain showed intense tracer uptake, suggestive of a malignant neoplasm, in a mass in her left cerebellar lobe. Our patient had experienced two years of occipital headache and movement disorder. Subsequently, magnetic resonance imaging was performed with contrast agent administration, showing a large subtentorial mass in her left cerebellar hemisphere, with compression and dislocation of the fourth ventricle. Metabolic data provided by positron emission tomography and morphological magnetic resonance imaging views were fused in post-processing, allowing a diagnosis of dysplastic gangliocytoma with increased glucose metabolism. Total resection of the tumor was performed and histological examination confirmed the diagnosis of Lhermitte-Duclos disease.

**Conclusions:**

Our case indicates that increased uptake of fluorine-18-fluorodeoxyglucose may be misinterpreted as a neoplastic process in the evaluation of patients with Lhermitte-Duclos disease, but supports the usefulness of integrated positron emission tomography-magnetic resonance imaging in the exact pathophysiologic explanation of this disease and in making the correct diagnosis. However, an accurate physical examination and exact knowledge of clinical data is of the utmost importance.

## Introduction

Lhermitte-Duclos disease (LDD), or dysplastic cerebellar gangliocytoma, is a rare tumor arising from the cerebellar cortex, first described clinically in 1920 by Lhermitte and Duclos [1]. The majority of cases are diagnosed in the third or fourth decade of life [1]. The common symptoms of this disease are due to the mass effect in the posterior cranial fossa, associated with cerebellar dysfunction, hydrocephalus and signs of increased intracranial pressure.

Till now, the exact origin of LDD, whether neoplastic, dysplastic or hamartomatous, is not well known; more than 200 cases have been reported in the literature [2] and these reports indicate that it is a benign neuroglial tumor with undefined prognosis. On histopathology, LDD is characterized by regional enlargement of the cerebellar stratum granulosum, an absence of the Purkinje cell layer and progressive hypertrophy of the granular cell neurons with increased myelination [3,4].

Magnetic resonance imaging (MRI) is considered to be the best modality imaging option for characterization of LDD by some authors [1,3,5] but the impact of functional imaging provided by nuclear medicine is recommended in other studies [2,5,6]. Various authors have described the potential role of molecular imaging provided by positron emission tomography (PET) and single-photon emission computed tomography in the metabolic evaluation of LDD, by using radiotracers such as fluorine-18-fluorodeoxyglucose (F18-FDG) [2,5,6], thallium-201 [5] and C11-methionine [7]. The potential added value of metabolic imaging lies in the possibility to calculate metabolic activity of a lesion by the measurement of the maximum standardized uptake value (SUV_max_), the ratio between the average radioactivity registered in a lesion and the administered amount of the same tracer [8].

The best therapeutic modality approach for this disease is represented by surgical intervention [9].

Here we describe a case of incidental LDD in a 37-year-old woman.

## Case presentation

A 37-year-old Caucasian woman came to our department to undergo a total-body PET-computed tomography (PET/CT) with F18-FDG for evaluation of a solitary lung node. Her family history was unremarkable. During clinical evaluation, she said she had been experiencing occipital headache and movement disorder for two years; therefore, in agreement with our patient, PET/CT of her brain, using three-dimensional acquisition, was performed after the total-body scan. Both PET/CT scans were performed after intravenous administration of 300 MBq of F18-FDG and fasting of six hours; image acquisition was performed with a hybrid Discovery ^16^ST PET-CT system (GE Medical System, Milwaukee, TN, USA).

No pathological uptake was detected corresponding to a solitary lung node with a 12 mm maximum transverse diameter and regular margins in the superior lobe of her right lung, in the CT images, nor in her other examined anatomic districts.

**Figure 1:**
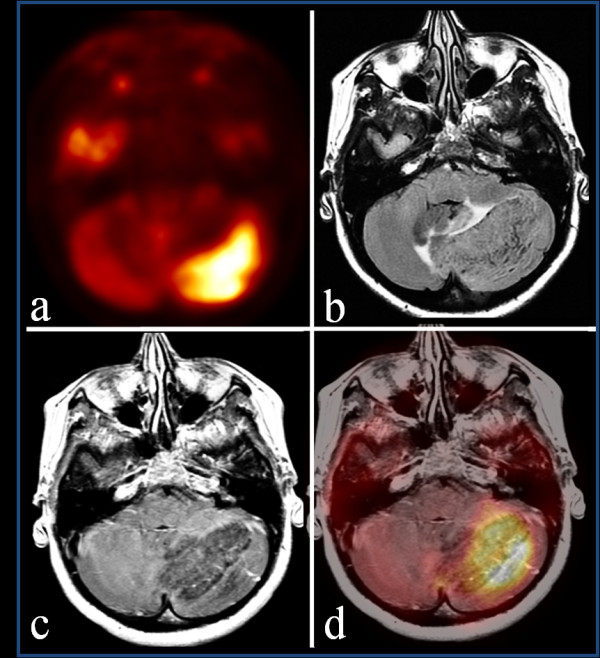
**Axial positron emission tomography view**. **(a) **Intense glucose metabolism in the left hemisphere of the cerebellum, corresponding to a hypointense and slightly enhancing area with **(b) **maximum diameter of 5 cm in axial T1-weighted post contrast view. **(c) **Axial T2-weighted fluid-attenuated inversion recovery images show the characteristic features of Lhermitte-Duclos disease: striations representing the folia of the cerebellum in association with hydrocephalus and dislocation of the fourth ventricle. **(d) **Axial fused positron emission tomography-magnetic resonance imaging views show the lesion with increased metabolism and intensity signal similar to that of normal grey matter.

A CT scan of her brain, performed before a cerebral PET scan, showed a hypodense mass in her left cerebellar hemisphere. Brain PET revealed intense and focal uptake of the tracer in the left hemisphere of her cerebellum, corresponding to the lesion discovered by CT, with an SUV_max _of 14, while the SUV_max _in the contralateral cerebellar hemisphere was 6.5 (Figure 1a).

Metabolic data were suggestive of a malignant condition. Four days later, MRI of her brain was performed. This revealed a slightly-enhancing, bulky mass in her right cerebellar hemisphere that measured 5 × 4 × 3 cm. On T1-weighted images, the lesion was hypointense and slightly enhancing after the administration of gadolinium. The T2-weighted fluid-attenuated inversion recovery (FLAIR) images showed the characteristic features of LDD: striations representing the folia of the cerebellum in association with hydrocephalus and dislocation of the fourth ventricle (Figure 1b,c). MRI was performed using a 3-T permanent magnet (GE Medical System).

We fused the PET images with MRI views of our patient. The comparative analysis of the images was achieved by using the software Advantage MRI-PET Fusion (GE Medical Systems), which is available on the system's workstation (Advantage Windows 4.4, GE Medical Systems) and allows simultaneous analysis of images. In particular, CT (obtained by co-registration with PET) and MR images were first aligned by identifying three or more pairs of corresponding landmarks to align the anatomical images. Subsequently, once the MR examination was sampled and adequately aligned with CT data, unnecessary CT data were discarded and, using the fusion software, were replaced by PET images. Post-processing thus yielded a single PET/MRI examination resulting from the fusion of the brain PET images with the brain MRI scans.

Image analysis of PET/MRI-fused images was performed by a nuclear medicine physician and a radiologist. The borders of the lesion were correctly fused and integrated PET/MRI views confirmed our suspicion that F18-FDG was taken up heterogeneously within the tumor, similarly to isointense bands of tissue in corresponding T2-weighted FLAIR images. Furthermore, by analysis of the PET/MRI images, we had confirmation of a lesion with similar metabolism and intensity signal to normal grey matter; the PET-only brain images suggested a hypothesis of a malignant neoplasm (Figure 1d).

Subsequently, total surgical resection of the lesion was performed and a histological examination confirmed the diagnosis of LDD. Histological examination of specimens revealed abnormal cerebellar cortical construction, showing a rather inversely arranged cell-layer construction. Large neurons with clear nuclear bodies and smaller neurons were present at the lateral sides of the folia.

## Discussion

On CT, the typical finding of LDD is a hypodense cerebellar mass, without contrast enhancement; on MRI, there is commonly a striated pattern of hypointensity on T1-weighted images and a hyperintensity on T2-weighted images alternating with isointense bands of tissue.

In the present case, the clinical presentation and MRI findings were in agreement with previously reported cases in the literature [3,5]; similarly to other previous studies, high level uptake of F18-FDG was observed [2,5,6]. The reasons for the high uptake of F18-FDG may be due to the overall high concentration of cerebellar cortical cells with high density, which can simulate a neoplasm. Taking this into account, we must underline that LDD metabolic features when using F18-FDG need to be correlate with traditional neuroimaging and, in the presence of an abnormal area of uptake in a cerebellar hemisphere, we cannot rule out the possibility of a non-malignant lesion.

MRI offers the possibility of correct identification of the disease, within a known pattern of typical findings. This suggested diagnostic tool as used in our case is linked to the feasibility of software that allows the fusion of metabolic imaging of PET with morphologic features of MRI, without any meaningful loss of diagnostic performance of either imaging modality [10].

New studies have focused on the possibility of evaluating MRI and PET in a single session: a recent paper examined the potential added value of fusion imaging PET/MRI in the evaluation of patients with cerebral tumors. In that study, patients were examined with 18F-FDOPA PET and by MRI; the authors concluded that fusion imaging may improve performance of both modalities, with the combination of the high sensitivity of PET with the high spatial resolution power and specificity of MRI. Combined 18F-FDOPA PET/MRI fusion provided precise anatomic localization of tracer uptake and well-labeled enhancing and non-enhancing tumor areas as well. It is important to underline that, in a small minority of cases, 18F-FDOPA activity identified a tumor not visible on MRI [11].

The possibility of integrated PET/MRI evaluation of the brain has a bigger value if we consider that new radiotracers have been developed in the last decade for the metabolic study of the brain. Recently, the feasibility of tracers such as F18-choline for characterization of low grade brain tumors [12] has been described, while another radiopharmaceutical, C11-methionine, has been employed in the evaluation of young patients with LDD. In this last study, the cerebellar lesions presented a high C11-methionine uptake (related to amino-acid hypermetabolism) in a slowly growing lesion while no uptake was detected in the resected tumors [7]. This fact suggests the feasibility of this examination in evaluating the efficacy of surgical treatment.

Considering the rapid development of new radiopharmaceuticals for molecular imaging of brain tumors [7,13], we can support the usefulness of integrated PET/MRI in diagnosis. Although MRI provides accurate morphological examination, the more important features of molecular imaging with PET could provide a better primary diagnosis of brain tumors, identify a molecular target for therapy [13] and finally assess the response to therapy.

## Conclusions

Although the exact pathophysiologic features of LDD are not well known, our case report indicates that morphofunctional imaging reflects an important aspect of this controversial disease: caution is needed in interpreting F18-FDG PET because the tracer can intensely accumulate in LDD, mimicking a malignant tumor. We suggest that correlative imaging with MRI and accurate knowledge of the clinical history of the patient are of the utmost importance. Simultaneous evaluation of both fused imaging modalities may provide new insight on the study of the brain and its changes in disease.

## Abbreviations

CT: computed tomography; FLAIR: fluid-attenuated inversion recovery; F18-FDG: F18-fluorodeoxyglucose; LDD: Lhermitte-Duclos disease; PET: positron emission tomography; MRI: magnetic resonance imaging; SUVmax: maximum standardized uptake value.

## Consent

Written informed consent was obtained from the patient for publication of this manuscript and any accompanying images. A copy of the written consent is available for review by the Editor-in-Chief of this journal.

## Competing interests

The authors declare that they have no competing interests.

## Authors' contributions

FC wrote the manuscript and interpreted the PET images; MB and CC were involved in the critical interpretation of the MRI. MZ, FC and ES elaborated fusion of the MRI and PET. MB was involved in the care of the patient, surgical intervention and English revision of the text. GG was involved in the figure, figure legend and in critical revision of CT images. OS critically revised the manuscript and figures. All authors read and approved the final manuscript.
